# The genome sequence of the Mother Shipton moth *, Euclidia mi* (Clerck, 1759)

**DOI:** 10.12688/wellcomeopenres.19098.1

**Published:** 2023-03-01

**Authors:** Douglas Boyes, University of Oxford and Wytham Woods Genome Acquisition Lab, Darwin Tree of Life Barcoding collective, Wellcome Sanger Institute Tree of Life programme, Wellcome Sanger Institute Scientific Operations: DNA Pipelines collective, Tree of Life Core Informatics collective, Peter W.H. Holland, Darwin Tree of Life Consortium

**Affiliations:** 1 UK Centre for Ecology and Hydrology, Wallingford, Oxfordshire, UK; 2 University of Oxford, Oxford, Oxfordshire, UK

**Keywords:** Euclidia mi, the Mother Shipton, genome sequence, chromosomal, Lepidoptera

## Abstract

We present a genome assembly from an individual male *Euclidia mi* (the Mother Shipton moth; Arthropoda; Insecta; Lepidoptera; Erebidae). The genome sequence is 2,320 megabases in span. Most of the assembly is scaffolded into 31 chromosomal pseudomolecules, including the assembled Z sex chromosome. The mitochondrial genome has also been assembled and is 15.6 kilobases in length. Gene annotation of this assembly on Ensembl identified 13,454 protein coding genes.

## Species taxonomy

Eukaryota; Metazoa; Ecdysozoa; Arthropoda; Hexapoda; Insecta; Pterygota; Neoptera; Endopterygota; Lepidoptera; Glossata; Ditrysia; Noctuoidea; Erebidae; Erebinae; *Euclidia*; *Euclidia mi* (Clerck, 1759) (NCBI:txid938167).

## Background

The Mother Shipton, *Euclidia mi* (Clerck, 1759) (= *Callistege mi*), is a day-flying moth in the family Erebidae, distributed widely across Europe and north into Scandinavia ( GBIF Secretariat, 2021). In the UK, the moth is most common in the south of England where it is frequently seen in May and June on chalk downland, heathland, woodland rides and flower-rich meadows. The adult moth is most active in sunny weather, but is a weak flyer and is usually seen flitting short distances before settling. The larvae have a series of orange, brown, black and white stripes running the length of the body and feed on clovers, trefoils, and other low-growing plants. Loss or reduction of larval prolegs has occurred in several members of the Erebidae including *E. mi*; retention of just three pairs of prolegs close to the posterior of the abdomen allows the larva to move by looping in a similar way to Geometridae larvae ( Byrne & Moyle, 2019). The species overwinters as a pupa.

The English common name, Mother Shipton, derives from the likeness to a face with exaggerated features traced out in profile by a white line on each forewing. The name seems to have originated in the late 18th century, since Moses Harris called the moth ‘the mask’ in the 1760s, but revised this to ‘the Shipton’ in the 1770s ( Thornton, 2006). ‘Mother Shipton’ herself was a reclusive prophet and seller of herbal remedies, born Ursula Sontheil in Knaresborough, Yorkshire, England, in 1488, and the subject of superstition and fear in her lifetime. Although Mother Shipton was already a historical figure when the moth was named, Harris was writing at a time of renewed interest in her life with the release of songs, pantomime and satire referring to her supposed prophecies ( Thornton, 2006). The cave where Mother Shipton lived can still be visited today. There is no evidence that the face-like wing markings on the moth are recognised as such by predators; the pattern is more likely a simple case of adaptation for crypsis amongst brown vegetation.

A high-quality genome sequence from *E. mi* and other day-flying moths will facilitate research into adaptations to a diurnal adult lifestyle, while comparison to data from other Erebidae will enable research into morphological evolution in this taxonomic family.

## Genome sequence report

**Figure 1.**
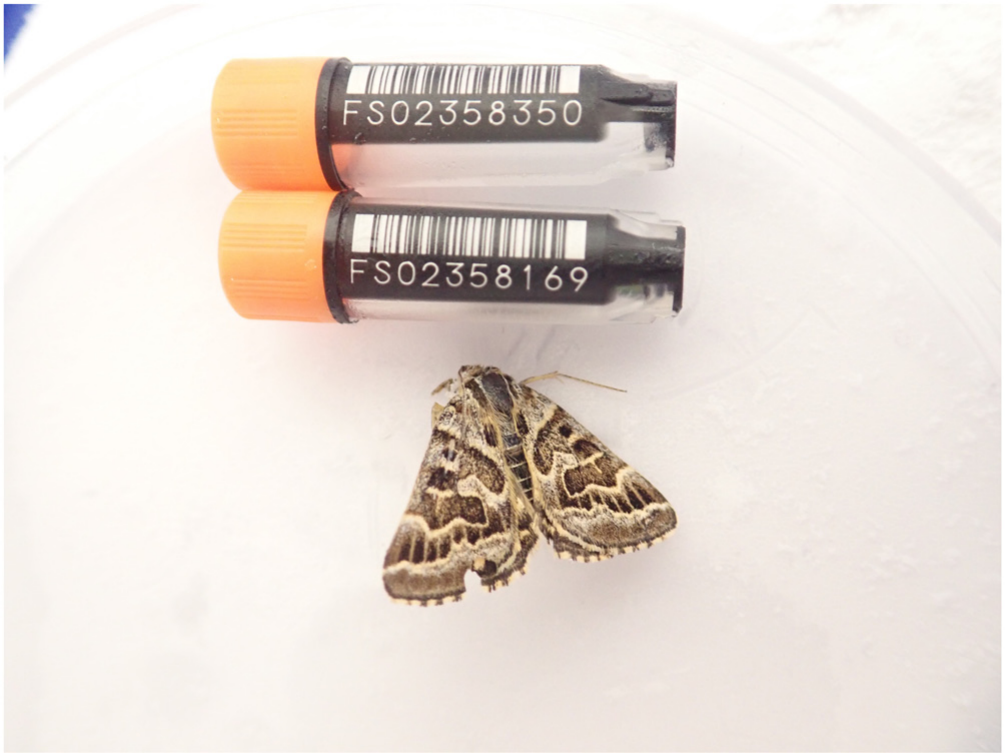
Photograph of the *Euclidia mi* (ilEucMixx1) specimen used for genome sequencing.

The genome was sequenced from one male *Euclidia mi* specimen ( Figure 1) collected from a grassland area of Wytham Woods (latitude 51.77, longitude –1.33). A total of 34-fold coverage in Pacific Biosciences single-molecule HiFi long reads was generated. Primary assembly contigs were scaffolded with chromosome conformation Hi-C data. Manual assembly curation corrected 119 missing joins or mis-joins and removed seven haplotypic duplications, reducing the assembly length by 0.92% and the scaffold number by 15.6%.

**Figure 2.**
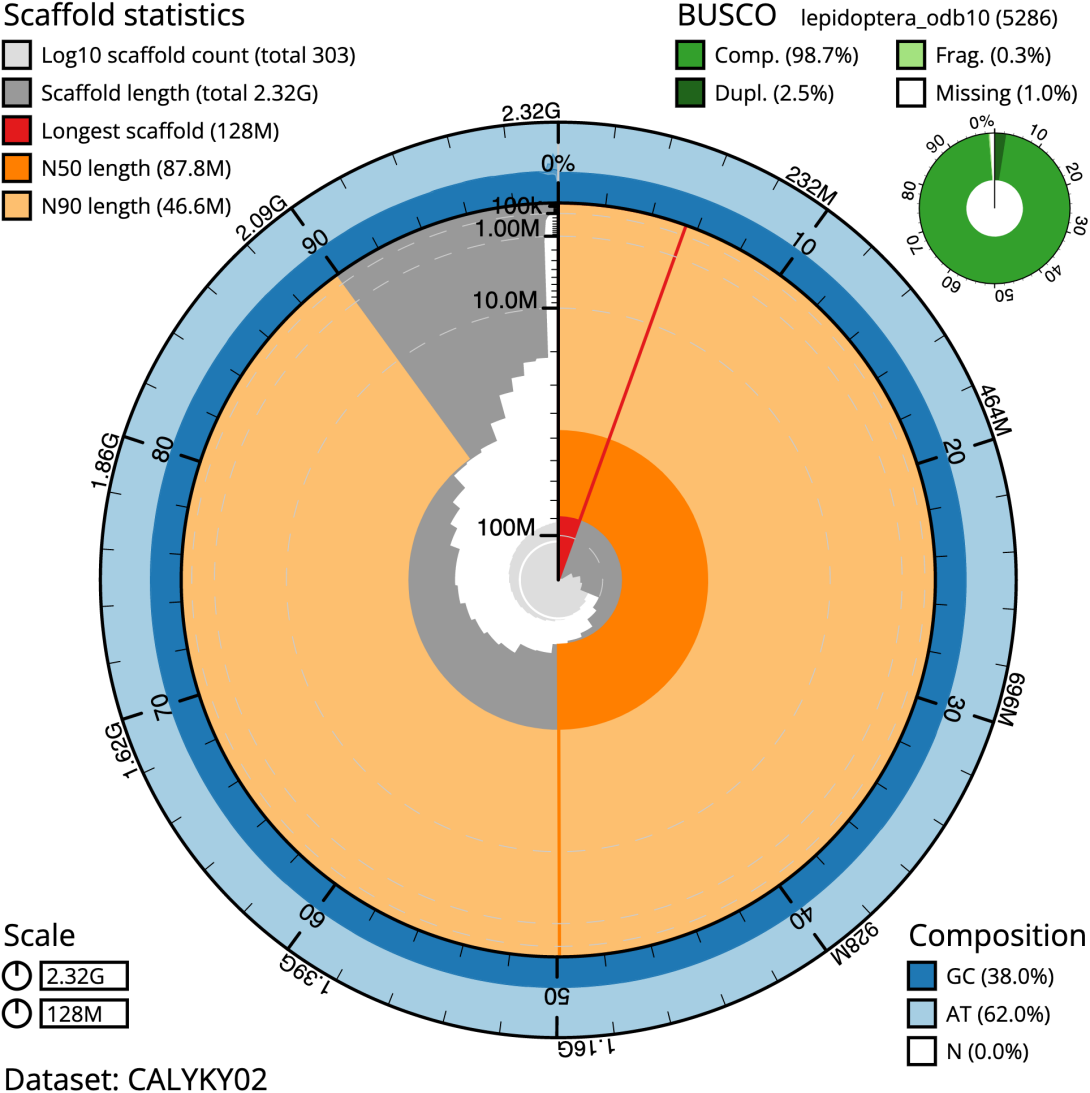
Genome assembly of *Euclidia mi*, ilEucMixx1.2: metrics. The BlobToolKit Snailplot shows N50 metrics and BUSCO gene completeness. The main plot is divided into 1,000 size-ordered bins around the circumference with each bin representing 0.1% of the 2,320,389,197 bp assembly. The distribution of scaffold lengths is shown in dark grey with the plot radius scaled to the longest scaffold present in the assembly (128,449,193 bp, shown in red). Orange and pale-orange arcs show the N50 and N90 scaffold lengths (87,750,765 and 46,627,851 bp), respectively. The pale grey spiral shows the cumulative sequence count on a log scale with white scale lines showing successive orders of magnitude. The blue and pale-blue area around the outside of the plot shows the distribution of GC, AT and N percentages in the same bins as the inner plot. A summary of complete, fragmented, duplicated and missing BUSCO genes in the lepidoptera_odb10 set is shown in the top right. An interactive version of this figure is available at https://blobtoolkit.genomehubs.org/view/ilEucMixx1.1/dataset/CALYKY01/snail.

The final assembly has a total length of 2,320.4 Mb in 303 sequence scaffolds with a scaffold N50 of 87.8 Mb ( Table 1). Most (99.4%) of the assembly sequence was assigned to 31 chromosomal-level scaffolds, representing 30 autosomes and the Z sex chromosome ( Figure 2– Figure 5; Table 2). The assembly has a BUSCO v5.3.2 ( Manni *et al.*, 2021) completeness of 98.7% (single 96.3%, duplicated 2.5%) using the lepidoptera_odb10 reference set. While not fully phased, the assembly deposited is of one haplotype. Contigs corresponding to the second haplotype have also been deposited.

**Table 1.** Genome data for *Euclidia mi*, ilEucMixx1.2.

Project accession data		
Assembly identifier	ilEucMixx1.2	
Species	*Euclidia mi*	
Specimen	ilEucMixx1	
NCBI taxonomy ID	938167	
BioProject	PRJEB53247	
BioSample ID	SAMEA7520660	
Isolate information	male ilEuMixx1: abdomen (PacBio); head/thorax (Hi-C) unknown sex ilEuMixx2 (RNA-Seq)	
Assembly metrics *		*Benchmark*
Consensus quality (QV)	63.2	*≥ 50*
*k*-mer completeness	100%	*≥ 95%*
BUSCO **	C:98.7%[S:96.3%,D:2.5%], F:0.3%,M:1.0%,n:5,286	*C ≥ 95%*
Percentage of assembly mapped to chromosomes	99.4%	*≥ 95%*
Sex chromosomes	Z chromosome	*localised homologous pairs*
Organelles	Mitochondrial genome assembled	*complete single alleles*
Raw data accessions		
PacificBiosciences SEQUEL II	ERR9836425–ERR9836428	
Hi-C Illumina	ERR9820271	
PolyA RNA-Seq Illumina	ERR9820272	
Genome assembly		
Assembly accession	GCA_944739405.2	
*Accession of alternate haplotype*	GCA_944738845.2	
Span (Mb)	2,320.4	
Number of contigs	1,835	
Contig N50 length (Mb)	2.8	
Number of scaffolds	303	
Scaffold N50 length (Mb)	87.8	
Longest scaffold (Mb)	128.5	
Genome annotation		
Number of protein-coding genes	13,454	
Number of non-coding genes	2,852	
Number of gene transcripts	23,514	

*Assembly metric benchmarks are adapted from column VGP-2020 of “Table 1: Proposed standards and metrics for defining genome assembly quality” from ( Rhie *et al.*, 2021). **BUSCO scores based on the lepidoptera_odb10 BUSCO set using v5.3.2. C = complete [S = single copy, D = duplicated], F = fragmented, M = missing, n = number of orthologues in comparison. A full set of BUSCO scores is available at https://blobtoolkit.genomehubs.org/view/Euclidia%20mi/dataset/CALYKY02/busco.

**Figure 3.**
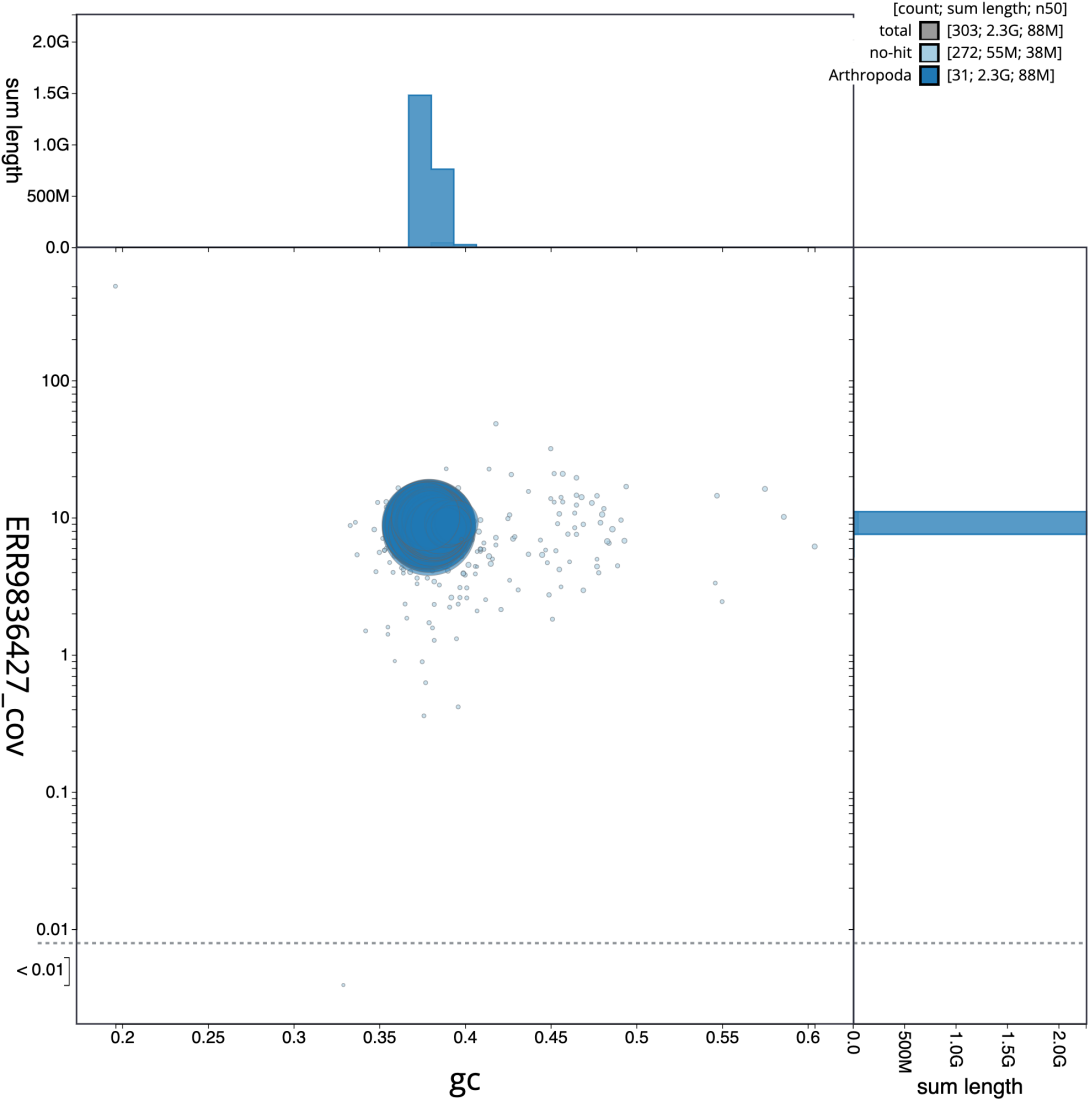
Genome assembly of *Euclidia mi*, ilEucMixx1.2: GC coverage. BlobToolKit GC-coverage plot. Scaffolds are coloured by phylum. Circles are sized in proportion to scaffold length. Histograms show the distribution of scaffold length sum along each axis. An interactive version of this figure is available at https://blobtoolkit.genomehubs.org/view/Euclidia%20mi/dataset/CALYKY02/blob.

**Figure 4.**
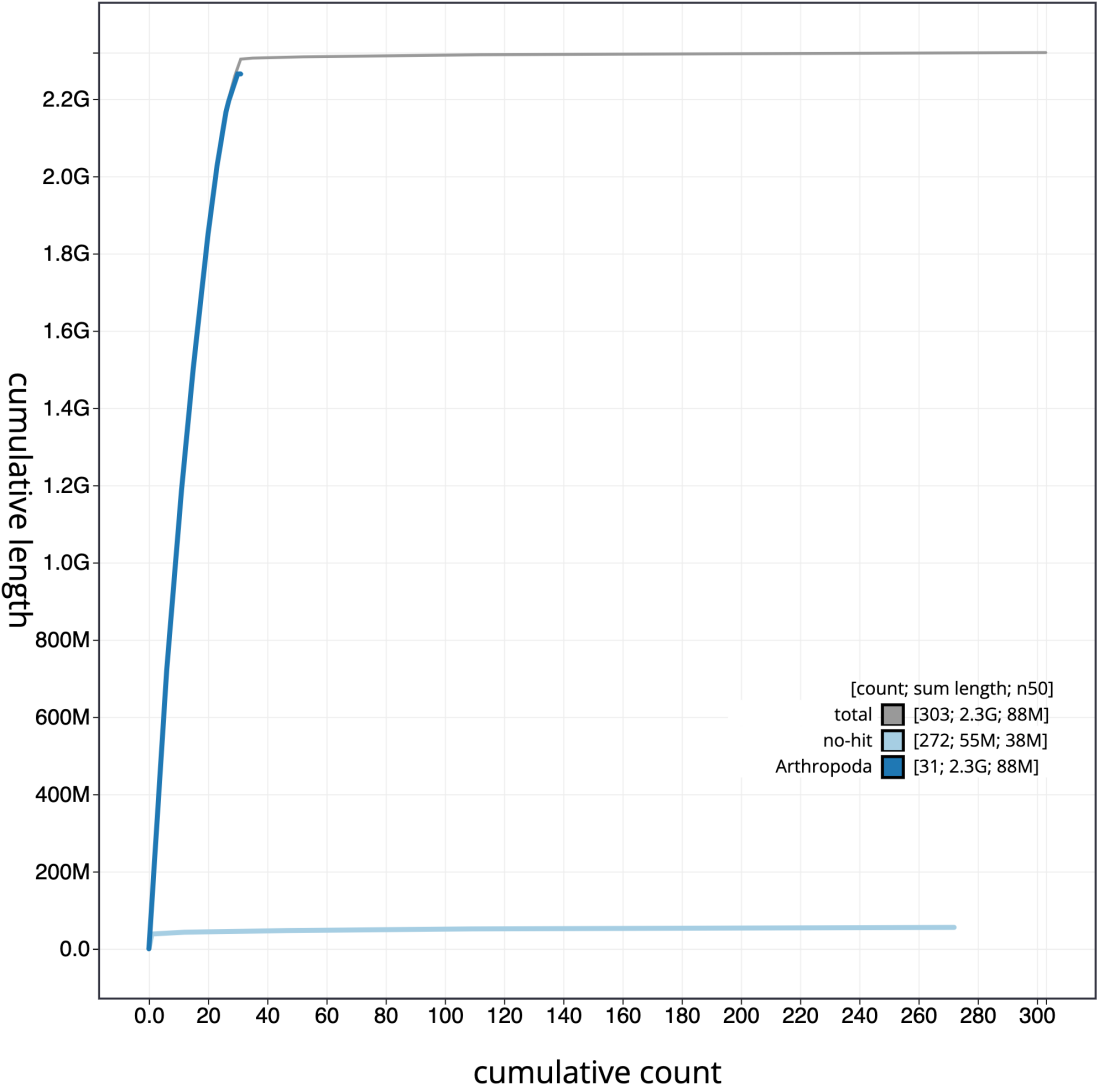
Genome assembly of *Euclidia mi*, ilEucMixx1.2: cumulative sequence. BlobToolKit cumulative sequence plot. The grey line shows cumulative length for all scaffolds. Coloured lines show cumulative lengths of scaffolds assigned to each phylum using the buscogenes taxrule. An interactive version of this figure is available at https://blobtoolkit.genomehubs.org/view/Euclidia%20mi/dataset/CALYKY02/cumulative.

**Figure 5.**
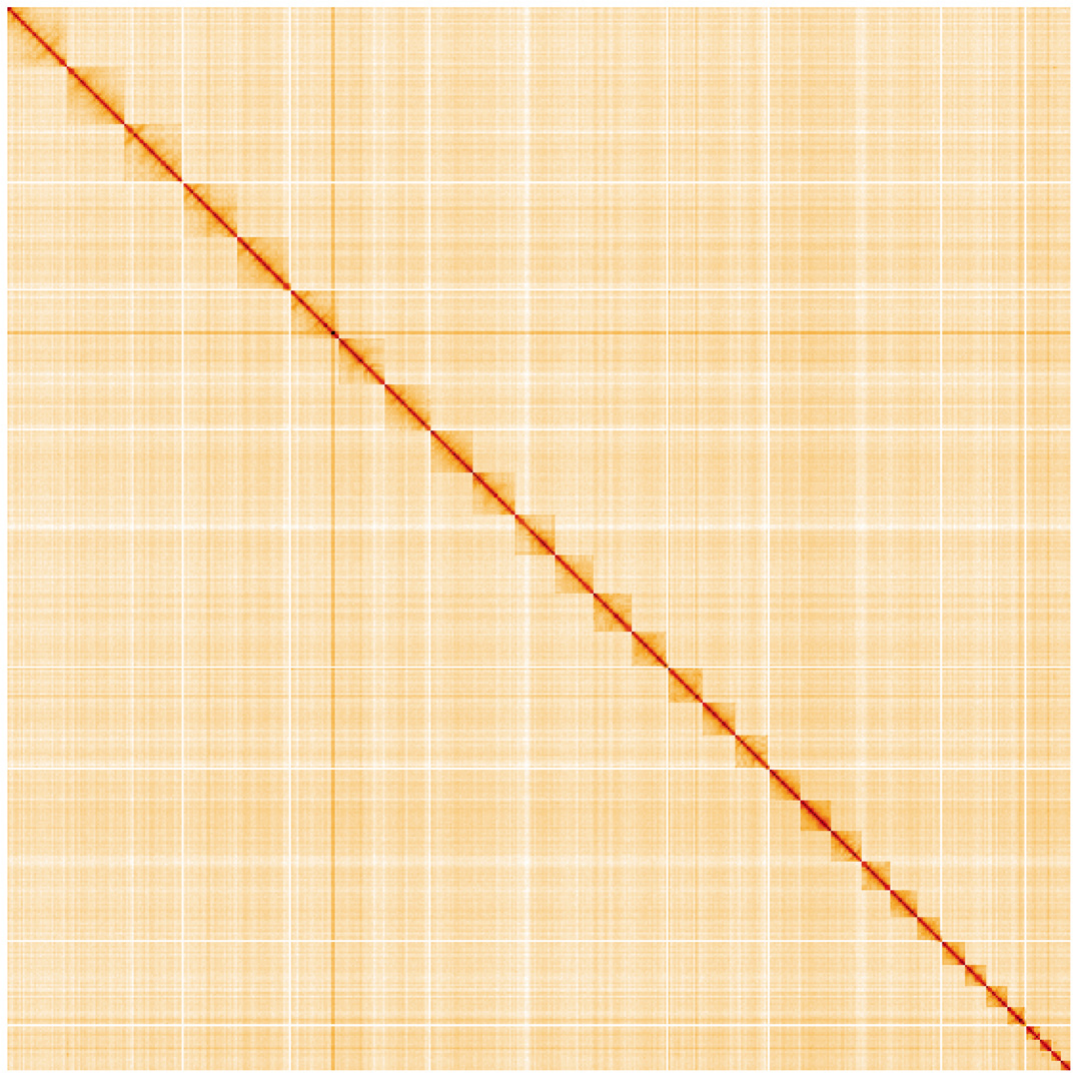
Genome assembly of *Euclidia mi*, ilEucMixx1.2: Hi-C contact map. Hi-C contact map of the ilEucMixx1.2 assembly, visualised using HiGlass. Chromosomes are shown in order of size from left to right and top to bottom. An interactive version of this figure may be viewed at https://genome-note-higlass.tol.sanger.ac.uk/l/?d=NtoBICfoT4Gs8i6iKcjyww.

**Table 2.** Chromosomal pseudomolecules in the genome assembly of *Euclidia mi*, ilEucMixx1.

INSDC accession	Chromosome	Size (Mb)	GC content (%)
OX123181.2	1	128.45	37.5
OX123182.2	2	126.51	37.5
OX123183.2	3	125.47	37.5
OX123184.2	4	117.95	37.5
OX123185.2	5	113.17	37.5
OX123186.2	6	111.88	37.5
OX123187.2	7	99.49	38
OX123188.2	8	96.65	37.5
OX123189.2	9	91.52	37.5
OX123190.2	10	89.33	37.5
OX123191.2	11	87.75	37.5
OX123192.2	12	82.69	38
OX123193.2	13	82.28	38
OX123194.2	14	76.58	37.5
OX123195.2	15	74.94	38
OX123196.2	16	73.01	38
OX123197.2	17	71.00	38
OX123198.2	18	68.46	38
OX123200.2	19	67.22	38
OX123201.2	20	62.27	38
OX123202.2	21	58.50	37.5
OX123203.2	22	54.24	38
OX123204.2	23	47.23	37.5
OX123205.2	24	46.63	38
OX123206.2	25	45.36	38.5
OX123207.2	26	38.25	38
OX123208.2	27	30.01	38.5
OX123209.2	28	25.79	39
OX123210.2	29	22.02	38.5
OX123211.2	30	21.31	39
OX123199.2	Z	67.21	37.5
OX123212.2	MT	0.02	19.5

## Genome annotation report

The *E. mi* GCA_944739405.1 genome assembly was annotated using the Ensembl rapid annotation pipeline ( Table 1; https://rapid.ensembl.org/Euclidia_mi_GCA_944739405.1/). The resulting annotation includes 23,514 transcribed mRNAs from 13,454 protein-coding and 2,852 non-coding genes.

## Methods

### Sample acquisition and nucleic acid extraction

Two *Euclidia mi* specimens (ilEucMixx1 and ilEucMixx2) were collected in Wytham Woods, Oxfordshire (biological vice-county: Berkshire), UK (latitude 51.77, longitude –1.33) on 30 May 2020 by netting. The specimens were collected and identified by Douglas Boyes (University of Oxford) and snap-frozen on dry ice.

DNA was extracted at the Tree of Life laboratory, Wellcome Sanger Institute (WSI). The ilEucMixx1 sample was weighed and dissected on dry ice with tissue set aside for Hi-C sequencing. Abdomen tissue was disrupted using a Nippi Powermasher fitted with a BioMasher pestle. High molecular weight (HMW) DNA was extracted using the Qiagen MagAttract HMW DNA extraction kit. HMW DNA was sheared into an average fragment size of 12–20 kb in a Megaruptor 3 system with speed setting 30. Sheared DNA was purified by solid-phase reversible immobilisation using AMPure PB beads with a 1.8X ratio of beads to sample to remove the shorter fragments and concentrate the DNA sample. The concentration of the sheared and purified DNA was assessed using a Nanodrop spectrophotometer and Qubit Fluorometer and Qubit dsDNA High Sensitivity Assay kit. Fragment size distribution was evaluated by running the sample on the FemtoPulse system.

RNA was extracted from head and thorax tissue of ilEucMixx2 in the Tree of Life Laboratory at the WSI using TRIzol, according to the manufacturer’s instructions. RNA was then eluted in 50 μl RNAse-free water and its concentration assessed using a Nanodrop spectrophotometer and Qubit Fluorometer using the Qubit RNA Broad-Range (BR) Assay kit. Analysis of the integrity of the RNA was done using Agilent RNA 6000 Pico Kit and Eukaryotic Total RNA assay.

### Sequencing

Pacific Biosciences HiFi circular consensus DNA sequencing libraries were constructed according to the manufacturers’ instructions. Poly(A) RNA-Seq libraries were constructed using the NEB Ultra II RNA Library Prep kit. DNA and RNA sequencing were performed by the Scientific Operations core at the WSI on Pacific Biosciences SEQUEL II (HiFi) and Illumina HiSeq 4000 (RNA-Seq) instruments. Hi-C data were also generated from tissue of ilEucMixx1 using the Arima v2 kit, and sequenced on the HiSeq X Ten instrument.

### Genome assembly

Assembly was carried out with Hifiasm ( Cheng *et al.*, 2021) and haplotypic duplication was identified and removed with purge_dups ( Guan *et al.*, 2020). The assembly was scaffolded with Hi-C data ( Rao *et al.*, 2014) using YaHS ( Zhou *et al.*, 2022). The assembly was checked for contamination and corrected using the gEVAL system ( Chow *et al.*, 2016) as described previously ( Howe *et al.*, 2021). Manual curation was performed using gEVAL, HiGlass ( Kerpedjiev *et al.*, 2018) and Pretext ( Harry, 2022). The mitochondrial genome was assembled using MitoHiFi ( Uliano-Silva *et al.*, 2022), which performed annotation using MitoFinder ( Allio *et al.*, 2020). The genome was analysed and BUSCO scores generated within the BlobToolKit environment ( Challis *et al.*, 2020). Table 3 contains a list of all software tool versions used, where appropriate.

**Table 3.** Software tools and versions used.

Software tool	Version	Source
BlobToolKit	4.0.7	Challis *et al.*, 2020
Hifiasm	0.16.1-r375	Cheng *et al.*, 2021
HiGlass	1.11.6	Kerpedjiev *et al.*, 2018
MitoHiFi	2	Uliano-Silva *et al.*, 2022
PretextView	0.2	Harry, 2022
purge_dups	1.2.3	Guan *et al.*, 2020
YaHS	yahs-1.1.91eebc2	Zhou *et al*. 2022

### Genome annotation

The Ensembl gene annotation system ( Aken *et al.*, 2016) was used to generate annotation for the *E. mi* assembly (GCA_944739405.1). Annotation was created primarily through alignment of transcriptomic data to the genome, with gap filling via protein to-genome alignments of a select set of proteins from UniProt ( UniProt Consortium, 2019).

### Ethics/compliance issues

The materials that have contributed to this genome note have been supplied by a Darwin Tree of Life Partner. The submission of materials by a Darwin Tree of Life Partner is subject to the Darwin Tree of Life Project Sampling Code of Practice. By agreeing with and signing up to the Sampling Code of Practice, the Darwin Tree of Life Partner agrees they will meet the legal and ethical requirements and standards set out within this document in respect of all samples acquired for, and supplied to, the Darwin Tree of Life Project. Each transfer of samples is further undertaken according to a Research Collaboration Agreement or Material Transfer Agreement entered into by the Darwin Tree of Life Partner, Genome Research Limited (operating as the Wellcome Sanger Institute), and in some circumstances other Darwin Tree of Life collaborators.

## Data Availability

European Nucleotide Archive: *Euclidia mi* (Mother Shipton). Accession number PRJEB53247; https://identifiers.org/ena.embl/PRJEB53247. ( Wellcome Sanger Institute, 2022) The genome sequence is released openly for reuse. The *Euclidia mi* genome sequencing initiative is part of the Darwin Tree of Life (DToL) project. All raw sequence data and the assembly have been deposited in INSDC databases. Raw data and assembly accession identifiers are reported in Table 1. Members of the University of Oxford and Wytham Woods Genome Acquisition Lab are listed here: https://doi.org/10.5281/zenodo.4789928. Members of the Darwin Tree of Life Barcoding collective are listed here: https://doi.org/10.5281/zenodo.4893703. Members of the Wellcome Sanger Institute Tree of Life programme are listed here: https://doi.org/10.5281/zenodo.4783585. Members of Wellcome Sanger Institute Scientific Operations: DNA Pipelines collective are listed here: https://doi.org/10.5281/zenodo.4790455. Members of the Tree of Life Core Informatics collective are listed here: https://doi.org/10.5281/zenodo.5013541. Members of the Darwin Tree of Life Consortium are listed here: https://doi.org/10.5281/zenodo.4783558.
