# Supplementary material for: The genome sequence of the Mother Shipton moth , Euclidia mi (Clerck, 1759)
Source: Wellcome Open Res. 2023 Mar 1;8:108. [Version 1] doi: 10.12688/wellcomeopenres.19098.1 (PMC10280027; doi:10.12688/wellcomeopenres.19098.1)
Supplement: Peer review history

# Peer review: Reviewer response for version 1

**DOI:** 10.21956/wellcomeopenres.21174.r59827

**Published:** 2023-06-21

**Authors:** Andrew J Mongue

**Affiliations:** 1 University of Florida, Gainesville, Florida, USA

The authors report the genome of *Euclidia mi*, a day-flying moth. The quality of the assembled genome is excellent, as expected from the Tree of Life programme and the manuscript is characteristically concise. Nevertheless, I appreciated that the authors included a greater background on biology and cultural history of this insect than most genome notes I have seen. 

 As another reviewer noted, the assembled genome is quite large for a ditrysian moth, not that I think the data are erroneous, merely that it is worth noting this represents a remarkably large genome for the order. Together with the fact that the number of annotated genes is in line with (if not a bit lower than) most Lepidoptera, it strongly suggests a high repeat content in the genome. It would be interesting to confirm this and explore which repeat families contribute the most to this massive genome, but given that such analyses tend to be beyond the scope of genome reports, I will not suggest it here.

Are sufficient details of methods and materials provided to allow replication by others?

Yes

Is the rationale for creating the dataset(s) clearly described?

Yes

Are the datasets clearly presented in a useable and accessible format?

Yes

Are the protocols appropriate and is the work technically sound?

Yes

Reviewer Expertise:

Population genomics, Lepidoptera, Hemiptera, genome assembly and annotation

I confirm that I have read this submission and believe that I have an appropriate level of expertise to confirm that it is of an acceptable scientific standard.

# Peer review: Reviewer response for version 1

**DOI:** 10.21956/wellcomeopenres.21174.r59822

**Published:** 2023-06-12

**Authors:** Alexander Mackintosh

**Affiliations:** 1 The University of Edinburgh, Edinburgh, Scotland, UK

This data note describes a genome assembly of the Mother Shipton moth ( *Euclidia mi*). It begins with a brief description of the ecology and biology of this species as well as the origin of its common name. The results section gives an overview of the data generated and also describes the genome assembly using relevant statistics and figures. Finally, the sampling, sequencing, assembly, and gene annotation steps are described in the Methods section.

 The report is concise yet contains enough information for readers to understand how the assembly has been generated.

 One interesting detail is that this is one of the largest lepidopteran genome assemblies (2.3 Gb) ever generated. The authors do not comment on this, but I expect that the transposable element content of the genome will be analysed and described in a future publication.

 This data note does not require any revisions. My only suggestion would be to include the contig N50 (2.8 Mb) in the main text rather than only in Table 1. Contig N50 is a more representative statistic of assembly contiguity than scaffold N50 (87.8 Mb), which is mostly determined by chromosome size.

Are sufficient details of methods and materials provided to allow replication by others?

Yes

Is the rationale for creating the dataset(s) clearly described?

Yes

Are the datasets clearly presented in a useable and accessible format?

Yes

Are the protocols appropriate and is the work technically sound?

Yes

Reviewer Expertise:

Population genomics

I confirm that I have read this submission and believe that I have an appropriate level of expertise to confirm that it is of an acceptable scientific standard.

# Peer review: Reviewer response for version 1

**DOI:** 10.21956/wellcomeopenres.21174.r56495

**Published:** 2023-05-10

**Authors:** Daniel Blande

**Affiliations:** 1 Biosafe - Biological Safety Solutions Ltd, Kuopio, Finland

In the article “The genome sequence of the Mother Shipton moth, *Euclidia mi* (Clerck, 1759) [version 1] the authors present a Data Note describing the sequencing, assembly and annotation of the *Euclidia mi* genome. The rationale for sequencing the genome is to facilitate research into day-flying moths and adaptations to a diurnal adult lifestyle. The sequencing methodology utilised PacBio HiFi long read sequencing for primary contig assembly, Illumina Hi-C for scaffolding and Illumina polyA RNASeq for use in annotation. A manual curation of the assembly was conducted to correct missing joins and mis-joins. The assembly statistics presented indicate a complete and high-quality assembly. The majority of the assembly has been assigned to 31 chromosomal level scaffolds. The raw data and assembly have been submitted to public repositories and the accession numbers for the raw data and assembly provided. The protocols and methods used are appropriate and technically sound.

 The tools used for the analysis and the software versions have been provided in Table 3 but the parameters have not been provided. Parameters differing from default values could be described in the text or added to the table to allow replication.

 At present there are two available accessions for the assembly ilEucMixx1.1 (GCA_944739405.1) which has been replaced by an updated assembly ilEucMixx1.2 (GCA_944739405.2). The latter assembly has a slightly larger size but it was not clear what had been changed between the two assemblies and occasionally which assembly was involved in a particular analysis. I have made specific comments below referencing the page number and section heading from the pdf version of the article:

- Page 3 - Genome Annotation Report. Please check the numbers in this section – 23,514 would represent total transcripts rather than transcribed mRNAs. The link in this section (at the time of review is to the assembly version ilEucMixx1.1) Has the annotation been performed on the ilEucMixx1.2 assembly and are the gene numbers the same between versions?
- Page 4, Table 1 – Do all Figures / statistics in this table refer to the ilEucMixx1.2 assembly? Consider if any statistics on the raw data would be useful here.
- Page 5 – Figure 2 – The link in the legend to an interactive plot is to an earlier version of the assembly ilEucMixx1.1 rather than the version shown in the figure which is ilEucMixx1.2.

Are sufficient details of methods and materials provided to allow replication by others?

Partly

Is the rationale for creating the dataset(s) clearly described?

Yes

Are the datasets clearly presented in a useable and accessible format?

Yes

Are the protocols appropriate and is the work technically sound?

Yes

Reviewer Expertise:

Bioinformatics, Genome Assembly, Genome Annotation.

I confirm that I have read this submission and believe that I have an appropriate level of expertise to confirm that it is of an acceptable scientific standard.
